# Component Identification and Analysis of Vesicular Fluid From Swine Infected by Foot-and-Mouth Disease Virus

**DOI:** 10.3389/fvets.2022.860978

**Published:** 2022-03-17

**Authors:** Ting Zhang, Bingzhou Lu, Bo Yang, Dajun Zhang, Xijuan Shi, Chaochao Shen, Huimei Cui, Xingguo Yuan, Dengshuai Zhao, Jinke Yang, Yu Hao, Xuehui Chen, Xiangtao Liu, Keshan Zhang, Haixue Zheng

**Affiliations:** State Key Laboratory of Veterinary Etiological Biology, National Foot-and-Mouth Disease Reference Laboratory, Lanzhou Veterinary Research Institute, Chinese Academy of Agriculture Science, Lanzhou, China

**Keywords:** swine, FMDV, vesicle fluid, identification and analysis, inflammation

## Abstract

Foot-and-mouth disease (FMD) is induced by FMD virus (FMDV) and characterized by fever and vesicular (blister-like) lesions. However, the exact composition of the vesicular fluid in pigs infected with FMDV remains unclear. To identify and analyze the components of the vesicular fluid in FMDV-infected domestic pigs, the fluid was collected and subjected to mass spectrometry. Further analyses were conducted using Gene Ontology (GO), Kyoto Encyclopedia of Genes and Genome (KEGG), and protein–protein interaction (PPI). Quantitative ELISA kit for TNF-α, and IFN-α, IFN-β, IL-6, IL-10, IL-1β, and IFN-γ were used to verify the mass spectrometry results. Results showed that 937 proteins were identified in the vesicular fluid from swine after FMDV infection, and bioinformatics analysis indicated that these proteins are related to the innate immune and inflammation pathways. The levels of cytokines involved in the disease-related pathways, tumor necrosis factors, and IL-6 in the fluid samples were significantly increased. This study identified and analyzed the composition of vesicular fluid in pigs after FMD infection for the first time and provided interesting information that help understand the infection and pathogenesis mechanism of FMD. These information will eventually contribute to the prevention and control of FMD.

## Introduction

Foot-and-mouth disease (FMD) is a highly contagious viral disease that affects both domestic and wild cloven-hoofed animals. The FMD virus (FMDV) is a member of the *Picornaviridae* family. FMD is endemic in many areas of the world. The disease is characterized by fever; vesicular (blister-like) lesions; and ulcers on the mouth, tongue, nostrils, muzzle, feet, and teats (1, 2). Transmission of FMDV can occur *via* respiratory, oral, or percutaneous route. The initial replication of the virus usually occurs at the site of entry, followed by its spread to the regional lymph nodes through the circulatory system. Serum specimens are useful for the detection of FMDV during viremia. The clinical signs of infection were reproduced in cattle exposed to this filtrate. In particular, cattle are more susceptible and show prominent vesicular lesions within the mouth and feet. Previous study, characteristic clinical manifestations of FMD in the laboratory, including whitening and vesicle formation of keratinocytes in the oral cavity and non-hair skin areas was observed (3).

Pathogenic microorganisms initiate innate immunity through the stimulation of pathogen pattern recognition receptors and can also cause the body to exert an inflammatory response (4). The recognition of the viral receptor binding sites and cell surface receptors is the first step of the viral infection pathway (5). This step helps determine the cell type and host tropism of the virus, which in turn affects the pathogenicity of the virus to different hosts. Mucosal epithelium (6) as the site of viral infection, oropharyngeal tonsil epithelial cells are the site of a large number of viral replications during the clinical stage of infection. Viremia occurs as early as 24 h post-exposure. Serum specimens are useful for detecting FMDV infection in case of viremia (7). Inflammation (8) is a complex defense response of living tissues to external injury. FMDV testing methods of sample and sample type to diagnostic specimens to FMDV surveillance in livestock (9), and there are many diagnostic methods for FMD, including laboratory testing *via* microscopy and ELISA (10). Nowadays, more medical fields are beginning to identify serum or tissue components for use as a biomarker for clinical diagnosis.

Infection elicits a rapid immune response. Infection and tissue injury are two main conditions that induce inflammation, thus triggering the infiltration of leukocytes and plasma proteins to the affected tissue site (11). During inflammation, local vasodilation and increased vascular permeability occurs through a series of reactions. This results in inflammation of the local tissues. The liquid component in the blood vessel. Cytokines (12) and proteins such as cellulose and various inflammatory cells enter the tissue through the blood vessel wall, causing an increase in local tissue components, which is manifested as edema. Highly expressed IL-1 cytokines play a key role in the pathogenesis of vesicular lesions by FMDV infection (13). The major IL-1 agonistic molecules are IL-1α and IL-1β. Studies have demonstrated that FMDV induces the secretion of caspase-1 and interleukin 1 beta (IL-1β) (14). IL-1β, TNF-α, and IL-6 are important indicators of inflammation. IL-1β is an important member of the IL-1 family. It has attracted much attention due to its important role in inflammation-related diseases. Strong proinflammatory activity, which can induce a variety of proinflammatory mediators. For local inflammation, IL-1β can lead to upregulation of adhesion molecules, thereby promoting the recruitment of lymphocytes. The local activation of IL-1β mediates the proinflammatory response. The central link leads to secondary inflammation (including IL-6). On the contrary, IL-6 systemically acts on the acute phase proteins produced by the liver, such as C-reactive protein, fibrinogen, and fibrinolysis enzyme activator inhibitor.

Therefore, to better understand the associated inflammation mechanism underlying FMD, the pathogenic behavior of FMDV should be analyzed. This study combined bioinformatics technology to explore the composition of vesicular fluid in FMDV infection. Kyoto Encyclopedia of Genes and Genome (KEGG) pathway mainly enriched the metabolic and innate immune pathways. We also tested the vesicular fluid and positive serum inflammatory cytokines related to understand natural immune-related factor expression in vesicular fluid.

## Materials and Methods

### Sample and Collection

Samples of vesicular fluid were collected from three FMDV-infected pigs. After 3 days of FMDV onset, we observed the clinical characteristics of vesicles. We collected the vesicular fluid, centrifuged it at 12,000 rpm at 4°C, and froze the supernatant at −80°C for future use. FMDV was verified in the vesicular fluid by RT-PCR, sequencing and sequence analysis. Serum samples from healthy pigs and diseased pigs were collected. Use a serum separator and allow sample to clot for 2 h at room temperature or overnight at 2–8°C.Centrifuge at approximately for 15 min at 1,000 × g.Assaay immediately or aliquot and store samples at −40°C. Avoid repeated freeze-thaw cycle.

### Mass Spectrometry

We used the AB SCIEX nano LC–MS/MS mass spectrometer. Protein lysis was first performed, and the supernatant was quantified using the BCA Protein Assay Kit and sodium dodecyl sulfate–polyacrylamide gel electrophoresis were used to determine the quantity and quality of proteins in the samples. The three samples were digested separately, and the digested products were analyzed by shotgun (LC-MS-MS) mass spectrometry. A total of three mass spectrometry analyses were performed. MS/MS spectra were searched using the MASCOT engine (Matrix Science, London, UK; v.2.2) against the UniProt Galagidae protein database (including 20,638 sequences, downloaded on v20200511). For protein identification, the following options were used: peptide mass tolerance = 20 ppm, MS/MS tolerance = 0.1 Da, enzyme = trypsin, missed cleavage = 2, fixed modification = carbamidomethyl (C), variable modification = oxidation (M), ion score > 20, and FDR <0.01 at peptide and protein levels.

### Bioinformatics Analysis

By going (Gene Ontology, http://en.wikipedia.org/wiki/GeneOntology) enrichment analysis between groups of protein function and KEGG database (said 49-year-old kyoko Encyclopedia of Genes and Genomes, http://www.kegg.jp/) to analyze and calculate the significance level of protein enrichment in each pathway, so as to determine the metabolic and transduction pathways that are significantly affected enrichment analyses were used to analyze the identified proteins. MaxQuant 1.5.5.1 software was used to search the corresponding database for the raw file of the mass spectrometry test and obtain the protein identification and quantitative analysis results. The significance enrichment analysis of GO functional annotations is to evaluate the significance level of protein enrichment degree of a certain GO functional item by Fisher's Exact Test. Omicsbean was used for the analysis of protein related biological information. Biological function was annotated and the signal pathway was enriched for the GO in the vesicular fluid and cross-compared to the KEGG.

### PPI Construction

Based on all datasets, after incorporating the data of the selected protein into the software using the online software String. To analyze the interaction between host proteins, a string database was used. Topological parameters and central measures of the network were calculated using a network analyzer tool in Cytoscape v.3.7.1. Pig protein–protein interaction analysis was also performed using the STRING database. The corresponding PPI network diagram was constructed.

### Quantitative ELISA

Using commercial kits, IL-6, IL-10, IL-1β, TNF-α, interferon (IFN)-α, IFN-β, and IFN-γ (Solarbio) were assayed. ELISA was performed according to the manufacturer's instructions and operated in sequence at 450-nm wavelength to determine the absorbance value of each well. A standard curve was constructed with the concentration of the standard substance as the abscissa and the absorbance value as the ordinate. The O.D. value of each standard substance and specimen should be subtracted from the BLANK hole, and the standard substance concentration should be used as the horizontal seat Standard, O.D. value as ordinate, using software to draw a standard curve, through the sample OD value can be found on the standard curve of its concentration. The data may be linearized by plotting the log of the cytokines concentrations vs. the log of the O.D. and the best fit line can be determined by regression analysis. This procedure will procedure an adequate but less precise fit of the data. If samples have been diluted, the con centration read from the standard curve must be muitipied by the dilution factor.

### Statistical Analyses

The GraphPad Prism5 software was used to process the data to evaluate the content of related cytokines in the vesicular fluid. The data are presented as means ± standard deviations. The *P*-value for statistical significance was <0.05 [^*^, *P* < 0.05 (significant); ^**^, *P* < 0.01 (highly significant)].

## Results

### Protein Identification

**Figure 1:**
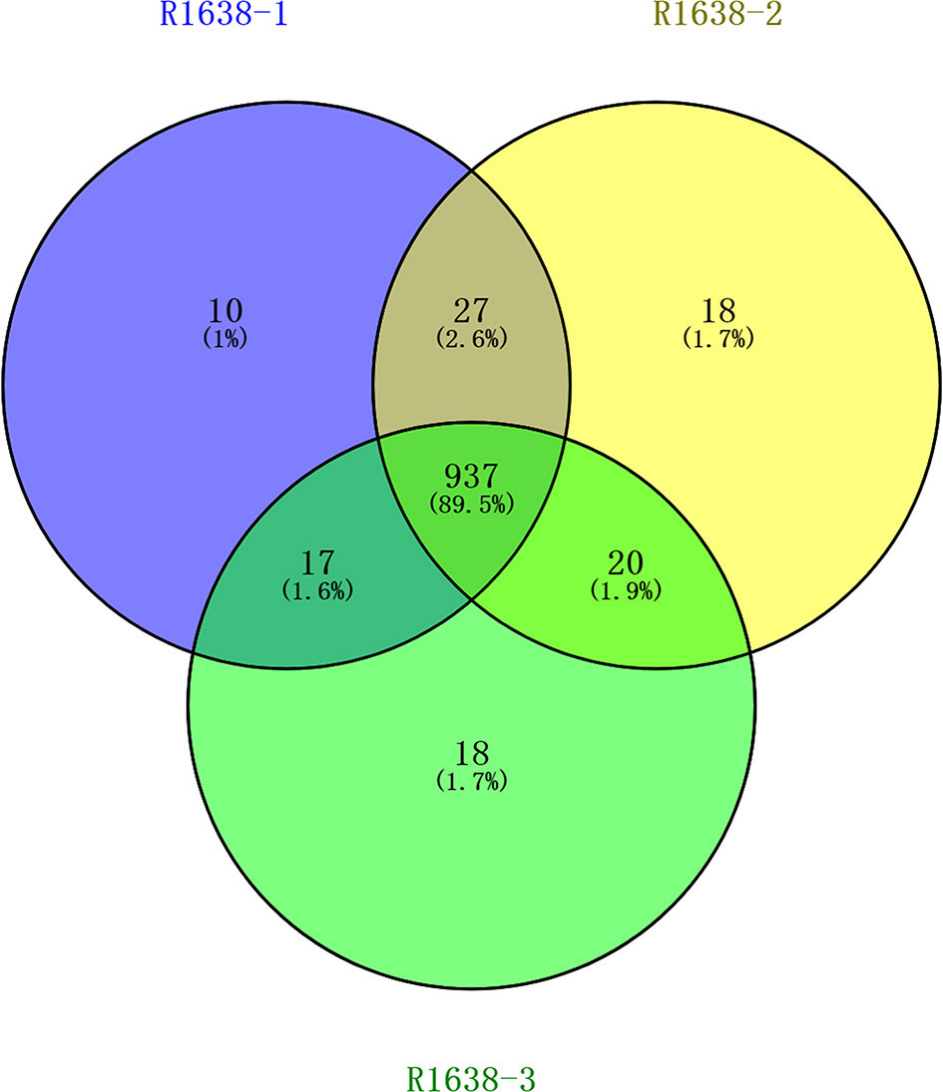
Venn diagram for serum protein identification. After preparation, the samples were lyophilized and incubated at 37°C for 16–18 h with 40 μl trypsin buffer. The hydrolysates were separated using capillary high-performance liquid chromatography and analyzed using Thermo Fisher mass spectrometry. MaxQuant 1.5.5.1 Searched the corresponding database, and finally obtained the results of protein identification and quantitative analysis.

Vesicular lesions in the mouth and on the feet are classic clinical signs of FMDV infection. After separation *via* capillary high-performance liquid chromatography, mass spectrometry was performed using Q Exactive mass spectrometer (Thermo Fisher). In total, 991, 1,002, and 937 proteins were identified in samples R1638-1, R1638-2, and R1638-3, respectively; the number of three sets of intersection proteins was 937 (Figure 1).

### Enrichment Analysis

**Figure 2:**
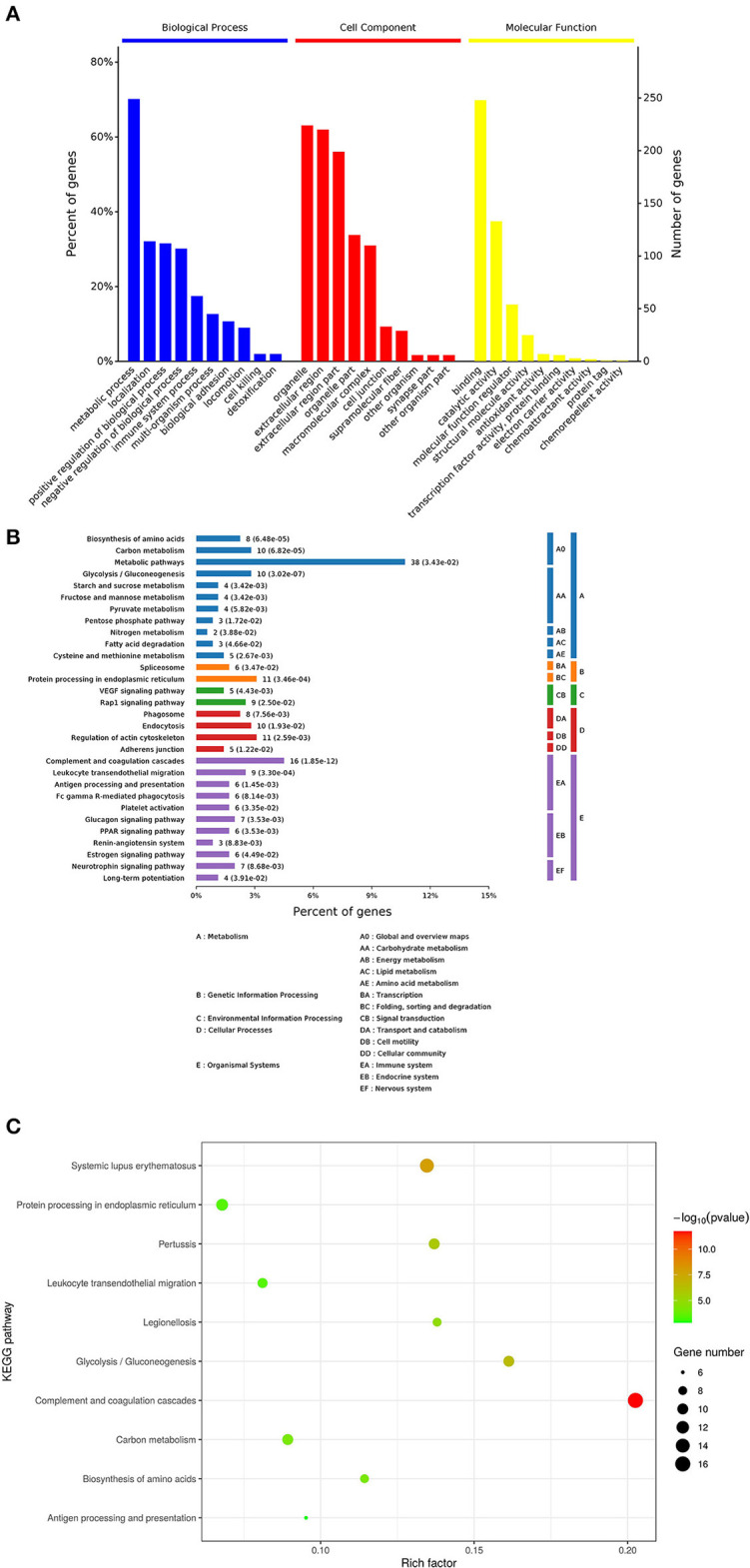
Enrichment analysis of the vesicular fluid by Proteomics. **(A)** By gene ontology, (http://www.geneontology.org/) in the GO database, the enrichment analysis of biological processes (BP), cell components (CC), and molecular functions (MF) is significant. The abscissa indicates the enrichment to GO functional classification. The figure shows the enrichment analysis results of BP, CC, and MF. **(B,C)** Kyoto Encyclopedia of Genes and Genomes (KEGG): the ordinate represents the top 10 significantly enriched KEGG pathways and the abscissa represents the enrichment factor of each KEGG pathway (rich factor ≤ 1). Enrichment factor indicates the proportion of the number of differentially expressed proteins involved in a certain KEGG pathway among all identified proteins; the color of bubbles indicates the significance of the enriched KEGG pathway.

GO enrichment analysis of proteins in each group was performed using Fisher (Fisher' Exact Test). The results showed that the most obvious upregulation of biological processes was that of the metabolic processes (Figure 2A) and the molecular function category are mainly enriched in cytokine activity, cytokine receptors. KEGG enrichment analysis revealed that the identified proteins were significantly enriched in pathways related to complement and coagulation cascades and protein processing in endoplasmic is high rich. The software Omicsbean was used to compare the distribution of each GO classification or classification or KEGG pathway in the target protein mass set and total body mass set, carry out the distribution of the target protein in the mass set, and carry out enrichment analysis of the GO annotation or KEGG pathway annotation for the target protein. The software R Version 3.5.1 was used to generate KEGG enrichment analysis bubble map. Viral proteins interacted significantly with cytokine and cytokine receptors (Figures 2B,C). Biological process is primarily involved in metabolic processes, biological process regulations, cellular processes, stimulation responses. Other biological processes and mainly plays the molecular functions such as binding enzyme regulation, catalysis, and so on. The results of KEGG pathway analysis revealed the complement activation and coagulation cascade, Activation of the complement system may be involved in innate immunity; therefore, the proteins related to this pathway were screened from the related pathways (Table 1).

**Table 1:** Kyoto encyclopedia of genes and genomes information on the vesicular fluid.

**Pathway ID**	**Pathway description**	**Count in** **gene set**	**FDR**
ssc04610	Complement and coagulation cascades	16	1.85E-12
ssc03320	PPAR signaling pathway	6	0.0415
ssc04370	VEGF signaling pathway	5	0.0475
ssc04010	MAPK signaling pathway	10	0.24
ssc04390	Hippo signaling pathway	6	0.289
ssc04152	AMPK signaling pathway	4	0.514
ssc04750	Inflammatory mediator regulation of TRP channels	3	0.596
ssc04620	Toll-like receptor signaling pathway	2	0.563
ssc04668	TNF signaling pathway	2	0.628
ssc04622	RIG-I-like receptor signaling pathwa	1	0.722

### PPI

**Figure 3:**
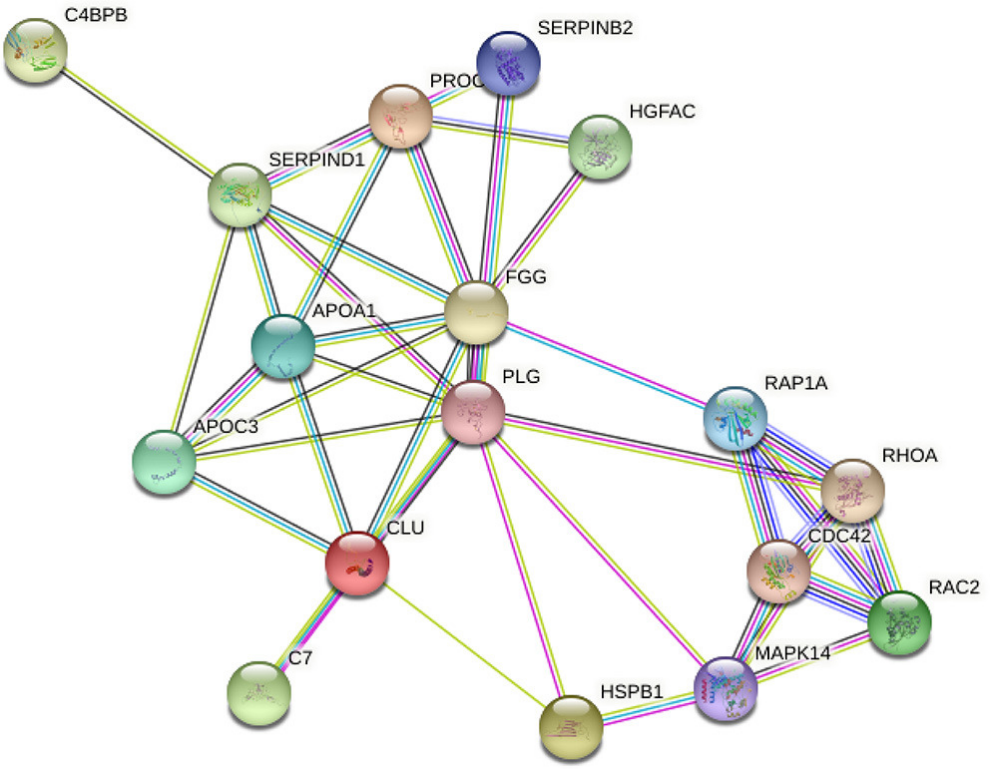
Correlation in protein abundance PPI using String. String (http://string-db.org/): the direct and indirect interactions between target proteins are found based on the information in the database.

This study constructed a PPI network diagram with the identified proteins of each group. The results are shown in Table 2. We know the inflammation an important cause of complement cascade and it promotes the formation of inflammatory process (15). Using the KEGG database, we screened the proteins related to complement, leukocyte migration, and MAPK signaling pathway, which was enriched in the complement activation, coagulation cascade, and peroxisome proliferator activated receptor signaling pathways. Therefore, we constructed several pathways in PPI network diagrams (Figure 3).

**Table 2:** Information on Kyoto encyclopedia of genes and genomes pathway-related proteins in the vesicular fluid.

**Protein ID**	**Gene name**	**KEGG pathway**
I3LJW2	FGG	Complement and coagulation cascades
Q5S1U1	HSPB1	VEGF signaling pathway
Q29549	CLU	Complement and coagulation cascades
F1RKY2	SERPIND1	Complement and coagulation cascades
I3LVS7	RHOA	Leukocyte transendothelial migration
Q007T2	CDC42	VEGF signaling pathway
F1S0J3	C4BPB	Complement and coagulation cascades
I3LRJ4	PROC	Complement and coagulation cascades
F1S8N1	HGFAC	Complement and coagulation cascades
F1SMJ1	C7	Complement and coagulation cascades
A0A287A9T4	RAC2	MAPK signaling pathway
F2Z5K3	RAP1A	Leukocyte transendothelial migration
K7GM40	APOA1	PPAR signaling pathway
P27917	APOC3	PPAR signaling pathway
A0A287AND4	SERPINB2	Complement and coagulation cascades
P06867	PLG	Complement and coagulation cascades
A0A287AX01	MAPK14	VEGF signaling pathway

### Increased Inflammation-Related Cytokines in Vesicular Fluid

**Figure 4:**
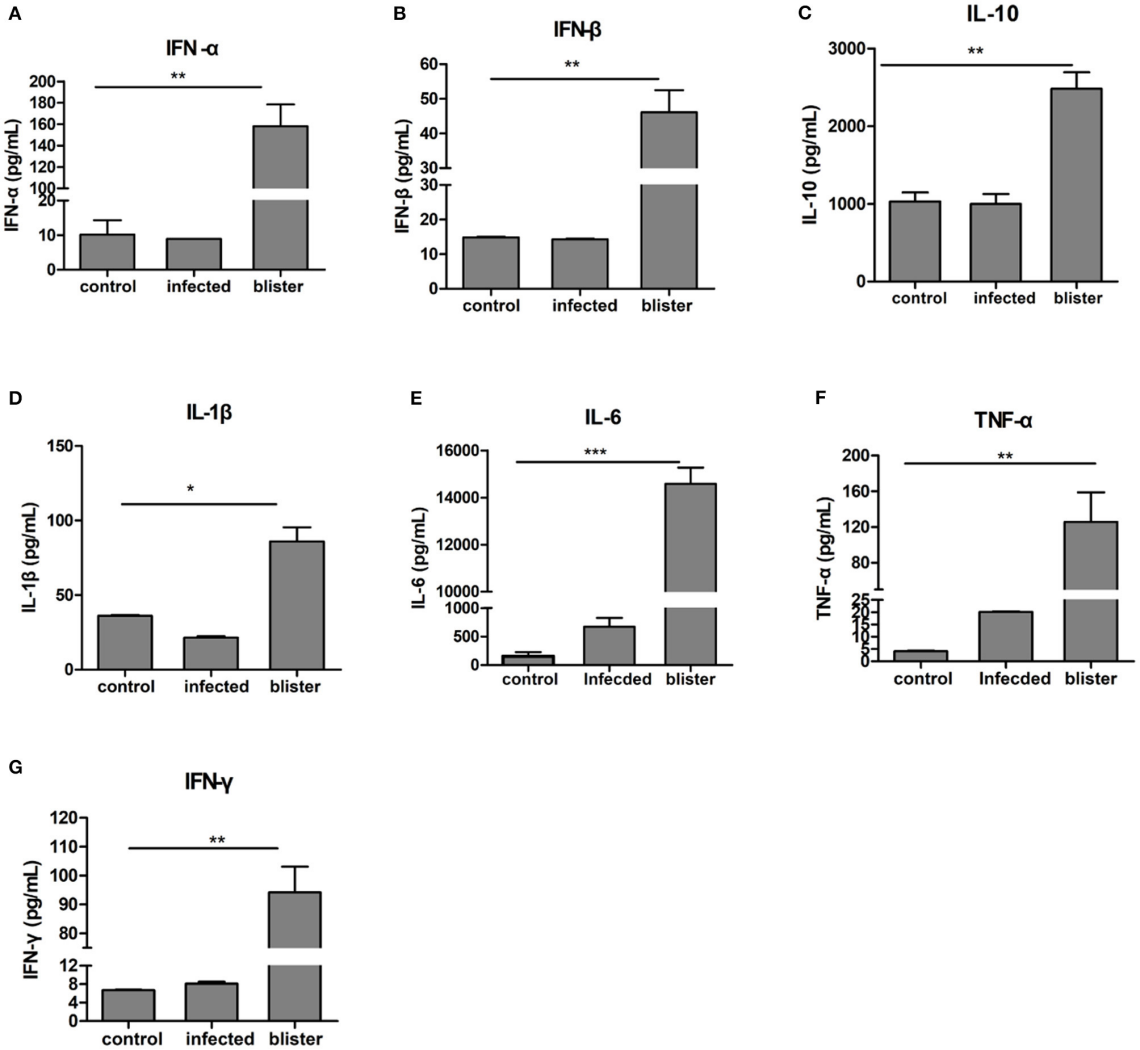
Differential expression of cytokines in the vesicular fluid. After centrifuging the aspirated vesicular fluid, the supernatant and healthy pig serum were absorbed, diluted 10 times using the standard of ELISA kit, and the absorbance value was obtained at 450 nm in strict accordance with the manufacturer's instructions; then the cytokine content was calculated. **(A,B)** ELISA, determination of the expression of type I interferon; **(C–F)** ELISA, determination of the expression of inflammatory cytokines in the vesicular fluid; **(G)** ELISA, determination of the expression of IFN-γ. T-test was used to calculate the significant difference, which is marked as *P < 0.05, **P < 0.01, ***P < 0.001 in the figure.

To evaluate the possible differences in the levels of inflammatory factors between the serum of healthy pigs and vesicular fluid of diseased pigs, we determined the level of inflammation-related cytokines in the vesicular fluid and serum of infected pigs and the serum of healthy pigs. Cytokines are a class of proteins active molecules involved in the communication among different cells of the immune system. In addition, many cytokines have regulatory functions outside the immune system. Among them, cytokines related to inflammation such as TNF-α, IL-1β, and IL-6 are the most important proinflammatory cytokines. We used ELISA to detect IFN-α, IFN-β, IFN-γ, IL-1β, IL- 6, IL-10, and TNF-α cytokines, (Figure 4). In the vesicular fluid and serum, the levels of proinflammatory cytokines IL-6 and TNF-α were higher compared with the controls; the levels of IL-6 and TNF-α were much higher in the vesicular fluid than in serum (Figure 4E). Thus, we hypothesized that IL-6 and TNF-α were involved in both the inflammatory response as well as the formation of skin lesions in FMD. It shows that inflammatory factors play an important role in vesicular fluid production, and it also implies that vesicular fluid can not only be used to isolate and identify viruses but also participate in the host's innate immune process.

## Discussion

The body can cause blisters through physical damage, such as burns (16). It can also pass some autoimmune diseases, such as Bullous pemphigoid (BP) (17). The inflammatory molecules present in serum and vesicular fluid such as cytokines and chemokines are hallmarks of the inflammatory response associated with BP. The body can be infected by pathogenic microorganisms, such as sepsis in the body (18). And FMDV can cause vesicular, combined with the formation mechanism of autoimmune disease blisters (19). The role of the cytokines and receptors of the IL-1 family in inflammation is well known (20). Data support the role of cytokines IL-1 and TNF-α in the pathogenesis of pemphigus vulgaris (21). IL-1β has emerged as a therapeutic target for an expanding number of systemic and local (22). We can find that the production of vesicular fluid in the body is related to the body's immune system. In most cases, autoimmune diseases cause subepidermal blistering. In addition to binding to the target antigen, autoantibodies also need to interact with factors of the innate immune system. Including the complement system and inflammatory cells, can cause vascular. Endothelial growth factors activate the VEGFR signaling pathway (23), triggering a network-like signal process and thereby promoting the growth, metastasis, and survival of vascular endothelial cells. Initially, VEGF was believed to promote the proliferation, survival, migration, invasion, formation of blood vessels, and increase in vascular permeability of vascular endothelial cells. The enhancement of vascular permeability induced by VEGFA is the basis for its important role in inflammation and other pathological environments. Histamine, bradykinin leukotriene, and substance P make endothelial cells contract rapidly, causing gaps between endothelial cells. Leukocyte IL-1β, TNF, interferon-γ, and hypoxia can cause endothelial cytoskeleton remodeling.

In this paper, it is found in the KEGG pathway of enrichment analysis that the body's metabolic pathways and complement activation and coagulation cascade pathways are significant. Similarly, we also found through verification that some cytokines such as IFN and TNF, chemokines, and TNF-α is a small molecule protein secreted by macrophages. TNF-α is mainly secreted by monocytes and macrophages. It not only plays a powerful role in killing or inhibiting tumor cells, promote the adhesion of neutrophils to endothelial cells, thereby stimulating local inflammation in the body. Acute inflammation is characterized by the release of a large number of molecules in the inflamed tissue and the release in the systemic circulation. Blood interleukins and cytokines are used to diagnose the biomarkers of inflammation caused by allergic reactions. This is similar to other diseases and pathogenic microorganisms that can cause inflammation. Such as BP is an autoimmune skin disease that can cause inflammation and the formation of subepidermal blisters, in which the vesicular fluid shows high levels of IL-6, IL-17 and other related cytokines (24). The components of vesicular fluid are also increasingly recognized as biomarkers for clinical diagnosis and routine research (25). Many studies have shown that FMDV VP1 protein is closely related to inflammation (26–28).

Prior to the development of the complement fixation test, FMDV infection was diagnosed primarily by clinical signs, the presence of vesicles on epithelial surfaces of the feet, mouth, nasal regions, and mammary glands. However, diagnosis based on clinical signs is complicated by the fact that other viral infections, swine vesicular disease virus, vesicular stomatitis virus, and vesicular exanthema of swine virus, may produce lesions which are indistinguishable from FMDV. Today, the detection of FMDV infections relies on the detection of FMDV-specific antibody (virus neutralization, antibody ELISA) or on the detection of the virus and/or viral components (virus isolation, antigen-capture ELISA), or reverse transcription polymerase chain reaction (RT-PCR). After domestic pigs are infected with FMDV, we can see blisters appear on the nasolabial region of pigs. The vesicular fluid in the blister can be used as a useful liquid tissue, reflecting the whole body and local microenvironment, and the cytokine level of the patient's vesicle fluid can also be dysfunction reflecting body skin damage (18). Hand and foot disease (29) that detection of blisters and vesicular fluid can reflect the changes of the body's cytokines. Therefore, the detection of the components in the vesicle fluid formed by FMDV infection has certain clinical guiding significance.

In summary, we identified vesicle fluid component and analyzed their function for the first time. It was generally observed to be at a higher level in the vesicular fluid, reflecting the fact that immunocytokines and immunomodulators are typically associated with a pro-inflammatory effect. ELISA results showed that IFN-α, IFN-β, IFN-γ and IL-1β, IL-10, IL-6, and TNF-α in vesicular fluid were significantly higher than in negative serum, suggesting that the changes of cytokines in microenvironment like vesicular fluid were more obvious. Therefore, vesicular fluid can replace serum as a biomarker. Studies on its components, immune-related cytokines and host proteins may be more conducive to the detection and prevention of FMDV, and provide ideas for clinical detection and control.

## Data Availability Statement

The original contributions presented in the study are included in the article/Supplementary Material, further inquiries can be directed to the corresponding author/s.

## Ethics Statement

The animal study was reviewed and approved by Lanzhou Veterinary Research Institute (Chinese Academy of Agriculture Science) Institutional Animal Care and conducted according to the AAALAC and the IACUC guidelines (License No. SYXK [GAN] 2014–003). Written informed consent was obtained from the owners for the participation of their animals in this study.

## Author Contributions

TZ, BY, HZ, and KZ conceived and designed the study. BL, DJZ, XS, CS, HC, XY, DSZ, JY, YH, XC, XL, KZ, and HZ wrote sections of the manuscript. All authors have read and agreed to the published version of the manuscript.

## Funding

This work was supported by grants from Natural Science Foundation of Gansu Province (20JR5RA582), National Key R&D Program of China (2021YFD1800300), and National Natural Science Foundation of China (31972684).

## Conflict of Interest

The authors declare that the research was conducted in the absence of any commercial or financial relationships that could be construed as a potential conflict of interest.

## Publisher's Note

All claims expressed in this article are solely those of the authors and do not necessarily represent those of their affiliated organizations, or those of the publisher, the editors and the reviewers. Any product that may be evaluated in this article, or claim that may be made by its manufacturer, is not guaranteed or endorsed by the publisher.

## Abbreviations

FMDV: Foot-and-mouth virus
IL: Interleukin
TNF-α: Tumor necrosis factor-α
IFN-γ: Interferon-γ
ELISA: Enzyme linked immunosorbent assay
OIE: the World Organization for Animal Health.
